# The biology of lysine acetylation integrates transcriptional programming and metabolism

**DOI:** 10.1186/1743-7075-8-12

**Published:** 2011-03-03

**Authors:** Jigneshkumar Patel, Ravi R Pathak, Shiraz Mujtaba

**Affiliations:** 1 Department of Structural and Chemical Biology, Mount Sinai School of Medicine New York, NY 10029 USA

## Abstract

The biochemical landscape of lysine acetylation has expanded from a small number of proteins in the nucleus to a multitude of proteins in the cytoplasm. Since the first report confirming acetylation of the tumor suppressor protein p53 by a lysine acetyltransferase (KAT), there has been a surge in the identification of new, non-histone targets of KATs. Added to the known substrates of KATs are metabolic enzymes, cytoskeletal proteins, molecular chaperones, ribosomal proteins and nuclear import factors. Emerging studies demonstrate that no fewer than 2000 proteins in any particular cell type may undergo lysine acetylation. As described in this review, our analyses of cellular acetylated proteins using DAVID 6.7 bioinformatics resources have facilitated organization of acetylated proteins into functional clusters integral to cell signaling, the stress response, proteolysis, apoptosis, metabolism, and neuronal development. In addition, these clusters also depict association of acetylated proteins with human diseases. These findings not only support lysine acetylation as a widespread cellular phenomenon, but also impel questions to clarify the underlying molecular and cellular mechanisms governing target selectivity by KATs. Present challenges are to understand the molecular basis for the overlapping roles of KAT-containing co-activators, to differentiate between global versus dynamic acetylation marks, and to elucidate the physiological roles of acetylated proteins in biochemical pathways. In addition to discussing the cellular 'acetylome', a focus of this work is to present the widespread and dynamic nature of lysine acetylation and highlight the nexus that exists between epigenetic-directed transcriptional regulation and metabolism.

## Introduction

DNA methylation and lysine modifications comprise major epigenetic processes on chromatin, which alter nucleosomal architecture leading to gene activation or repression [1-3]. Dynamic post-translational modifications (PTMs) occurring in the proximity of a gene promoter are one of the hallmarks of epigenetic regulation of gene expression [4]. Although an individual lysine residue may undergo mutually exclusive multiple PTMs, including acetylation, methylation, neddylation, ubiquitynation and sumoylation, multiple lysines of a single protein can undergo diverse modifications [5,6]. Functionally, these site-specific PTMs, which are established during transcriptional programming, impart flexibility to regulate cellular processes in response to diverse physiological and external stimuli. PTMs impact functional capabilities of a protein, thus validating the notion that biological complexities are not restricted only by the number of genes [7]. To elucidate the functional consequences of a single PTM or combinatorial PTMs occurring on chromatin, the histone code hypothesis proposes to integrate the gene regulatory ability of a site-specific histone modification within its biological context [8,9]. In quintessence, a site-specific PTM serves as a mark to recruit a chromatin-associated protein complex(es) that participates in controlling gene activity, thereby, regulating cell fate decisions [10]. For instance, within chromatin, depending on the site and degree of the modification, lysine methylation can cause either gene activation or repression; lysine acetylation on histones is associated with chromatin relaxation contributing to gene activation; and the biochemical outcome of lysine ubiquitynation or sumoylation is dynamic turnover of proteins. In addition, although the role of methylation in modulating non-histone proteins, including transcription factor activity, is only beginning to be understood, acetylation of transcription factors can affect their DNA-binding ability, stability, nuclear translocation and capacity to activate target genes [7,11].

Accumulating studies focusing on model systems of viral infection and the DNA-damage response have supported the role for lysine acetylation in enhancing molecular interactions between transcription factors and the transcriptional machinery on a gene promoter, leading to modulation of a specific downstream target [3,12-14]. Mechanistically, addition of an acetyl group to a lysine residue alters the positive charge of the ε-amino group, thereby impacting electrostatic properties that prevent hydrogen bonding and generating a circumferential hydrophobic milieu. Subsequently, this alteration of charge could facilitate acetylation-directed molecular interactions. Historically, almost four decades ago, acetylation of histones was first speculated to be involved in gene transcription. However, it was not until 1996 that one of the first lysine acetyltransferase (KAT), HAT-A from *Tetrahymena*, was cloned and characterized [15]. Very recently, combinatorial approaches with high-affinity acetyl-lysine antibodies, mass spectrometry (MS) and stable-isotope amino-acid labeling (SILAC) techniques detected almost 2000 acetylated proteins in the cell [16,17]. Further, the functional implications of each of these PTMs will have to be determined; one of the major tasks will be to distinguish a dynamic acetyl mark(s) specific for a pathway from a set of pre-existing global marks. Studies demonstrate that lysine acetylation can initiate molecular interplay leading to at least one of the two biochemical outcomes: 1) recruit co-activator complexes via conserved modular domains such as bromodomains; 2) engage co-repressor complexes through lysine deacetylases (KDACs) [18,19]. Published studies have utilized trichostatin A (TSA) or other KDAC inhibitors to highlight the biochemical significance of acetylation [20,21]. Long-term therapeutic aspirations stem from the pharmacological inhibition of KDACs that provides clinical benefits in models of human disease. Histone deacetylation reverts the electrostatic characteristics of chromatin in a manner that favors gene repression. Interestingly, a recent genome-wide chromatin immunoprecipitation analysis revealed preferential association of KDACs with active genes, suggesting that KDACs do not simply turn off genes, but rather function to fine-tune gene expression levels [22].

Cellular-wide proteomic analyses on protein acetylation revealed a large number of acetylated proteins, mostly enzymes involved in intermediary metabolism in the cytoplasm as well as the mitochondrion [16,23,24]. These findings support a larger role of acetylation extending beyond the nucleus mainly toward the regulation of metabolic enzymes by at least two mechanisms: 1) acetylation-mediated modulation of metabolic enzymatic activity; and 2) influencing their protein stability [17,25,26]. Given the frequent occurrence of metabolic dysregulation in human diseases, including diabetes, obesity and cancer, acetylation could play a pivotal role in the progression of these diseases. Particularly in cancers, it is well known that the transcriptional functions of the tumor suppressor p53 are affected by alterations in tumor-cell metabolism [27,28]. Because enzymes that catalyze acetylation are also transcriptional co-activators, which coordinate with transcription factors in regulating gene expression--underscoring the integration of transcription with metabolism--such enzymes present potential therapeutic targets. The overall goal of this review is to highlight the most recent advances in the field of acetylation biology that could spark new perspectives and illuminate novel research avenues.

## The versatile and conserved nature of Lysine acetylation

**Figure 1:**
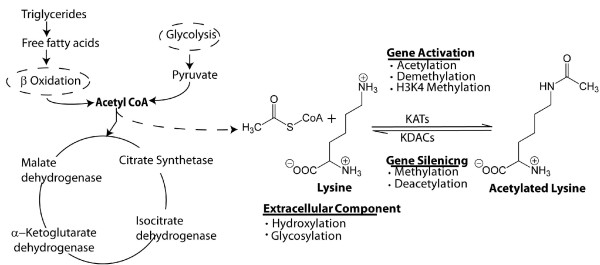
**A lysine residue targeted by co-factors and enzymes mediating epigenetic events that regulate cellular processes**.

The versatile nature of the amino acid lysine is exhibited not only by its ability to undergo a wide range of epigenetic modifications implicated in chromatin signaling networks but also by its indispensable structural role in extracellular matrices. The ε-amino group participates in hydrogen bonding and acts as a general base in catalysis. This unusual chemical plasticity within a lysine residue eliminates steric hindrance to allow histone-modifying enzymes that are central to transcriptional regulation to perform acetylation and methylation as well as subsequent deacetylation and demethylation (Figure 1).

Lysine acetylation was initially identified in histones, so KATs and KDACs were referred to as histone acetyltransferases (HATs) and deacetylases (HDACs), respectively. There are three major groups of KATs: Gcn5-related N-acetyltransferases (GNATs); E1A-associated protein of 300 kDa (p300; KAT3A) and CBP (KAT3B); and MYST proteins [10,29]. Known KDACs are divided into classes I, II and IV and the sirtuin family (also known as class III KDACs). In humans, there are KDAC1, -2, -3, and -8 (class I); KDAC4, -5, -6, -7, -9, and -10 (class II); and KDAC11 (class IV) [30]. There are seven members of the sirtuin family in humans (SIRT1-7) [22,31]. Wang and colleagues [22] recently analyzed the genome-wide localization of KDACs and their KAT counterparts in human immune cells. Surprisingly, KDACs were not recruited to silenced gene promoters. Instead, both KATs and KDACs were enriched on inactive promoters that had methylation of histone H3 at lysine 4 (H3K4me) and were also enriched on active promoters. The occurrence of KDACs on promoters imply deacetylation, which will prevent RNA polymerase II from binding to genes that are standing by to be activated but should not yet be switched on. For instance, KDACs might also contribute to the removal of undesired basal acetylation. Collectively, these results indicate a major role for KDACs in the maintenance of gene activation.

**Figure 2:**
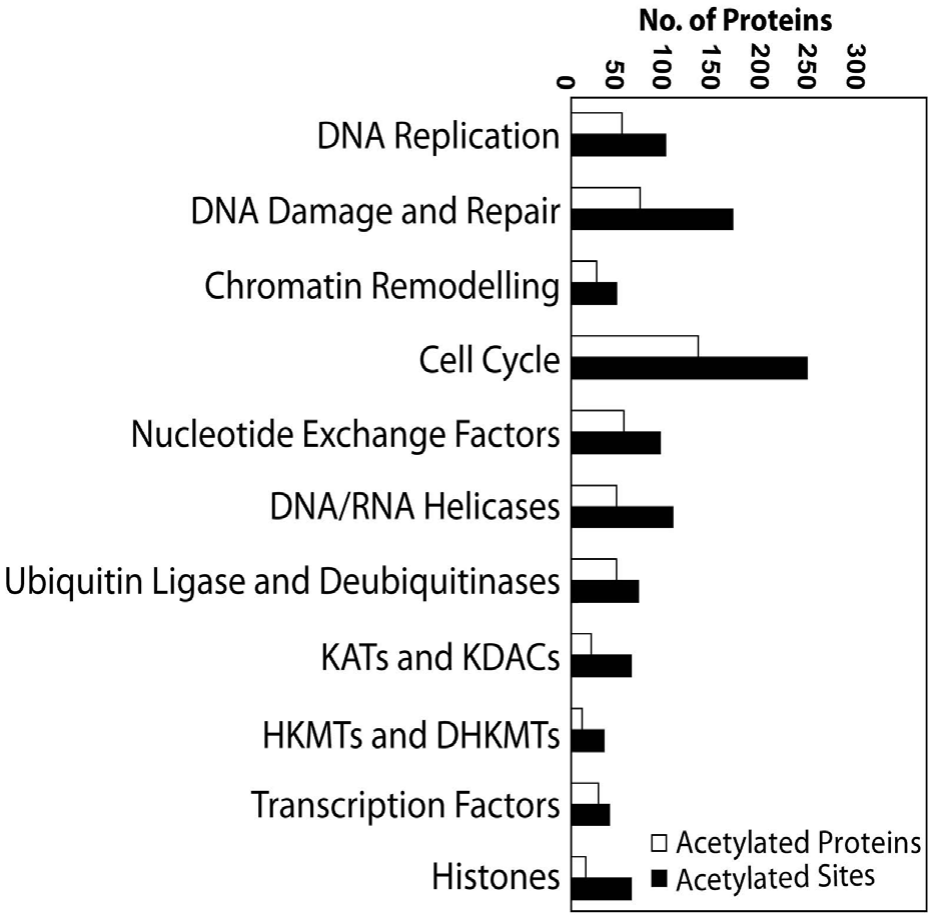
**Graphic and qualitative representation of the functional distribution of acetylated proteins in a human cancer cell line**.

Several studies have described acetylated proteins from mouse liver, human leukemia cells, and more recently from human liver cells [17,23,25]. Out of the 1047 acetylated proteins from human liver, 135 overlapped with 195 acetylated proteins from mouse liver. However, only 240 acetylated proteins were common between the human liver and leukemia cells, suggesting that differential profiles of acetylated-proteins could be physiologically relevant and also cell-type-dependent [16]. In leukemic cells, using high-resolution MS, 3600 lysine acetylation sites were identified on 1750 proteins [16]. Our analysis of the supplementary data from that study using the functional annotation clustering tool DAVID 6.7 showed that the lysine-acetylated proteins can be categorized into more than 500 functional clusters (Additional file 1**Table S1**), thereby extending our knowledge of the cellular events that are regulated by acetylation [32,33]. This functional annotation clustering tool identifies related genes or proteins by measuring the similarity of their global annotation profiles based on the hypothesis that if two genes have similar annotation profiles, they should be functionally related. Using this rationale, the method identifies broader gene groups whose members share major biological features. Based on the output generated by this tool the acetylated proteins were determined to be involved in the regulation of numerous processes such as mRNA processing, proteolysis, GTP binding, stress responses, regulation of cell death, immune system development, neuron development and differentiation, and regulation of the protein kinase cascade. Interestingly, more than 500 acetylated unique proteins with multiple acetylation sites were categorized as being involved in chromatin-templated processes (Figure 2). Functional annotation clustering revealed the acetylated proteins to be involved in regulation of Parkinson's disease, Huntington's disease, Alzheimer's disease and glycogen storage disease. An important functional cluster that emerged from our analyses included more than 50 acetylated proteins involved in various types of cancers. KAT3B, retinoblastoma and tumor suppressor p53 figured prominently in the list of proteins implicated in human diseases (Additional file 2**Table S2**).

Lysine acetylation is a prevalent modification in enzymes that catalyze intermediary metabolism, and our analyses extended the scope of this regulation [17]. Lysine acetylated proteins are involved in the metabolism of carbohydrates, lipids, nucleotides, amino acids, secondary metabolites, and xenobiotics. Acetylation also regulates the relative activities of key enzymes controlling the course of glycolysis versus gluconeogenesis, and the branching between the citrate cycle and glyoxylate bypass. This modulation within metabolic pathways is directed by a KAT and KDAC pair whose expression levels are synchronized according to growth conditions. Reversible acetylation of metabolic enzymes ensures rapid cellular responses to environmental changes through prompt sensing of cellular energy status and flexibly altering reaction rates.

Until very recently, lysine acetylation was known only in eukaryotic cellular processes, although its existence in prokaryotes was predicted. Substantiating this idea, very recently, it was shown that reversible lysine acetylation regulates acetyl-coenzyme A synthetase activity in *Salmonella enterica *[25]. Acetylation of metabolic enzymes that depend on a carbon source indicates that acetylation may mediate adaptation to various carbon sources in *S. enterica*, which has only one major bacterial protein acetyltransferase, Pat, and one nicotinamide adenine dinucleotide (NAD+)-dependent deacetylase, CobB. To determine whether and how lysine acetylation globally regulates metabolism in prokaryotes, Zhao *et al*. determined the overall acetylation status of *S. enterica *proteins under fermentable glucose-based glycolysis and under oxidative citrate-based gluconeogenesis [17]. Moreover, those authors demonstrated that key metabolic enzymes of *S. enterica *were acetylated in response to different carbon sources concomitantly with changes in cell growth and metabolic flux.

In addition to the epigenetic modifications on lysines that occur in chromatin, collagen contains hydroxylysine, which is derived from lysine by lysyl hydroxylase. Furthermore, allysine is a derivative of lysine produced by the action of lysyl oxidase in the extracellular matrix and is essential in crosslink formation and stability of collagen and elastin. Similarly, *O*-glycosylation of lysine residues in the endoplasmic reticulum or Golgi apparatus is used to mark certain proteins for secretion from the cell. Interestingly, lysine is metabolized in mammals to give acetyl-CoA, via an initial transamination with α-ketoglutarate, which is then utilized as a substrate by KATs. Bacterial degradation of lysine yields cadaverine by decarboxylation. Although histidine and arginine are also basic amino acids, they are not subjected to PTM as is lysine. Taken together, these findings signify that mechanisms regulating metabolism may be evolutionarily conserved from bacteria to mammals. Furthermore, characterization of acetylation-mediated regulatory mechanisms in bacteria would offer new perspectives in advancing our understanding of many hitherto unknown biological processes. In the remainder of this article, we concentrate on a few important proteins that require acetylation to execute their functions and which have been widely investigated but still remain a subject of intense of biochemical research.

## The acetylation-directed transcriptional program

### Acetylation engendered chromatin milieu on gene regulation

**Figures 3:**
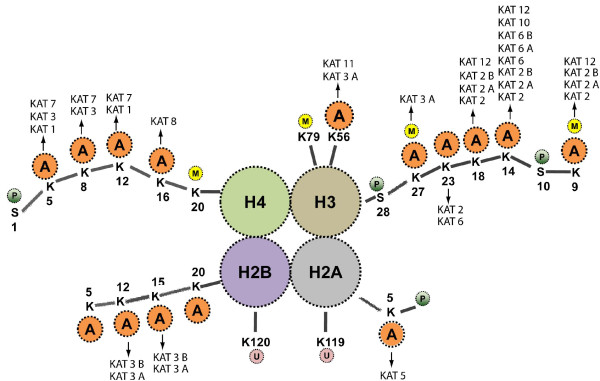
**Lysine acetyltransferases involved in acetylating histone proteins**. In chromatin, A denotes acetylation; M, methylation; P, phosphorylation and U, ubiquitination.

The earliest explanation of histone acetylation was in the physicochemical context that nucleosomes and chromatin impose a barrier to transcription. Subsequently, it became apparent that lysine acetylation neutralizes the positive charges on histone tails (Figure 3), relaxing their electrostatic grip on DNA to cause nucleosomal remodeling that exposes transcription factor binding sites [34]. Furthermore, because acetylated lysine moieties on histone tails could serve as recruitment sites for bromodomain-containing cofactors or reversal of charge by KDACs, this not only suggested an additional mechanism for KAT-directed gene activation [35], but also established that acetylation, like phosphorylation, creates a new scaffold to recruit proteins to the nucleosome. Notably, in charge-neutralization models, acetylation of multiple lysine residues--that is, hyper-acetylation--on a single histone tail should produce a stronger effect than mono-acetylation. By contrast, in bromodomain-recruitment models, in which adjacent amino acids determine specificity, a single lysine residue on a histone tail is paramount, and possible hyper-acetylation of the entire tail may not be expected to contribute further to recruitment [36]. It is also possible that for a specific lysine residue, both modalities of acetylation may be physiologically relevant and apply in different circumstances, as suggested from *in vitro *studies of H4-K16 acetylation [37,38]. Moreover, adding to the complexities of acetylation, it cannot be ruled out that acetylated moieties may also recruit KDACs to regulate tightly and temporally transcriptional activation, as mentioned above.

### The puzzling cross-talk on chromatin between post-translationally modified sites

#### Acetylation versus methylation

A growing body of evidence suggests that independent of their proximity, co-existing histone modifications can have synergistic or antagonistic effects on gene expression. This also highlights that epigenetic marks are not deposited or recognized in isolation but comprise a complex and interrelated collection of modifications at adjacent residues on a given nucleosome of a gene promoter. The correlation between different histone modifications is particularly clear for acetylation of histone H3 (Figure 3) and methylation of histone H3 at lysine 4. This is consistent with the observed co-localization of these marks, which show correlated distribution patterns both on a chromosome-wide scale during X inactivation and over the coding regions of individual genes [39,40]. These correlations may arise due to physical links between histone-modifying enzymes such that they are co-recruited to the same loci. Both KMT2A/MLL1, a lysine methyltransferase (KMT) that can generate H3K4me marks [41], and Chd1, the chromatin remodeler that is subsequently recruited by this methyl mark, associate with KAT activities [42,43], whereas the LSD1 complex that removes some of these methyl marks contains the lysine deacetylases KDAC1 and KDAC2 [44]. However, the interaction could also arise due to the mechanism of action of these enzymes. For example, the SET domain of KMT2A has a preference for acetylated substrates [41].

#### Acetylation versus phosphorylation

Multiple cellular processes are associated with histone phosphorylation: DNA damage induces phosphorylation on serine 139 of H2A (H2AS139p) [10,45]; transcription, upon mitogenic stimulation, on H3S10p [10]; mitosis on H3S10p and H3S28p; apoptosis, depending on the stimulus used, on H4S1p, H3S10p, H2BS32p, and H2AS32p [9,10]. Serum stimulation induces the PIM1 kinase to phosphorylate pre-acetylated histone H3 at the FOSL1 enhancer [46]. The adaptor protein 14-3-3 binds the phosphorylated nucleosome and recruits KAT8/MOF, which triggers acetylation of H4K16 [47]. Histone crosstalk generates the nucleosomal recognition code composed of H3K9ac/H3S10p/H4K16ac that determines the nucleosome platform for binding of the bromodomain protein BRD4 [48,49]. Recruitment of the positive transcription elongation factor b (P-TEFb) via BRD4 induces the release of the promoter-proximal paused RNA polymerase II and increases its processivity. Thus, the single phosphorylation on H3S10 at the FOSL1 enhancer triggers a cascade of events which activates transcriptional elongation. Increasing evidence also show that several types of modifications are linked and, in particular, one modification may influence the presence of a nearby modification [9,10,50]. This has been demonstrated for H3K14ac and H3S10p on the histone H3 tail, as well as, for H3S10p and H3K9me on the same tail [51]. Whereas the first pair of modifications has been coupled to activation of gene expression, increasing evidence indicates that H3K9me results in decreased H3S10p and is thereby responsible for silencing.

### Impact of acetylation on the functioning of transcription factors

**Figures 4:**
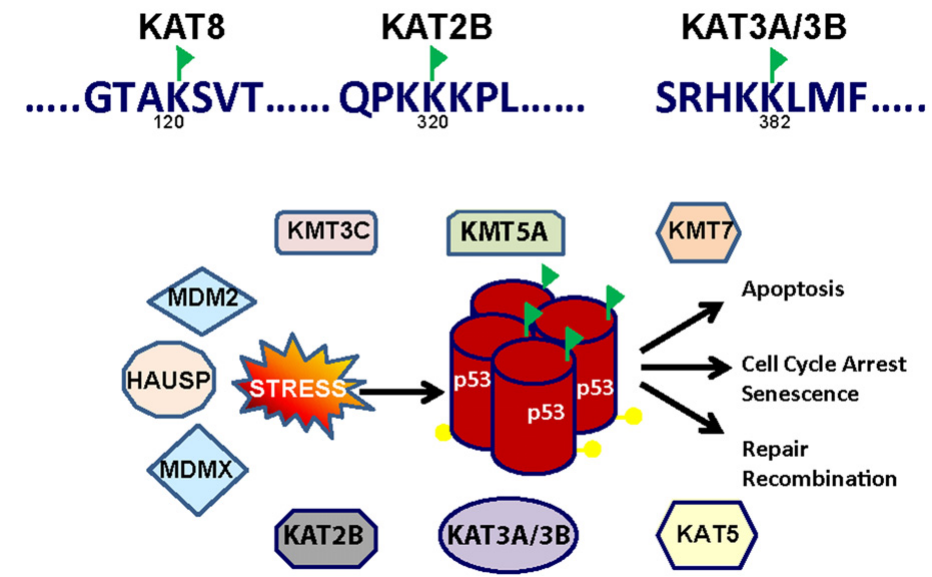
**Lysine acetyltransferases responsible for mediation of the stress response by p53**. The green flag stands for acetylation and the yellow ball-stick represents phosphorylation of p53 during stress.

The tumor suppressor protein p53 functions as a transcription factor to orchestrate a transcriptional program that controls many target genes during a wide variety of stress responses [52,53]. After sensing a genetic aberration (such as DNA damage), the sequence-specific DNA-binding ability of p53 enables it to directly participate in controlling target gene transcription to alter cellular responses. In addition to being a DNA-binding transcription factor, p53, following stress, undergoes extensive PTMs to enhance its function as a transcription factor in controlling cellular decisions that could culminate in cell-cycle arrest, senescence or apoptosis [52,53] (Figure 4). Interestingly, p53 has a short half-life; however, depending on the nature of the stress, the cell type and the profile of PTMs rendered, p53 will promptly execute a transcriptional program beneficial to the cell [54]. In addition, through protein-protein interactions, p53 can bind to and recruit general transcription proteins, TAFs (TATA-binding protein-associated factors), to induce transcription of target genes [55-57]. Recent experiments have shown that p53 can also engage with KATs, including KAT3B, KAT3A, KAT5/Tip60 and KAT2B/PCAF to the promoter region of genes [58-60].

In 1997, Gu and Roeder showed that acetylation of p53 on its C-terminal lysines by KAT3A/3B is crucial for p53 activation during DNA damage [59,60]. Subsequently, the biochemical significance of p53 acetylation was established in cancer cell lines under various genotoxic stresses and oncogenic Ras activation that lead to the interaction of acetylated p53 with KAT3A and PML [61-63]. In parallel, it was proposed that p53, upon activation, undergoes a wave of phosphorylation on its N-terminus that precludes its degradation by MDM2 and concomitantly brings in KAT3A/3B to acetylate the C-terminal end of p53 [64,65]. At this stage, KAT3A/3B-catalyzed p53 acetylation was implicated in enhancing p53's DNA-binding ability, nuclear localization and co-activator recruitment functions [11]. Later, KAT2B was also shown to acetylate lysine 320 of human p53 and lysine 317 of mouse p53 [62]. Recent identification of the p53K120ac site in the p53 DNA-binding region supports a direct role for acetylation in p53- DNA interactions. Lysine 120 on p53 is acetylated by KAT5 and/or KAT8/MOF [58,66]. Taken together, the impact of acetylation on p53 function can be attributed to the inhibition of nonspecific DNA-binding, recruitment of bromodomain-containing co-activators for target-gene activation, and modulation of KDAC activity to regulate target-gene activation. Nevertheless, the most puzzling aspect of p53 PTMs remains to be clarified: the mechanism by which acetylation of the C-terminus of p53 produces mutually exclusive lysine methylation or ubiquitination. Recent studies revealed that p53K370, K372 and K382 can also be methylated, indicating cross-regulation between acetylation, methylation and ubiquitynation. One recent study speculates that p53K372me recruits KAT5 through its chromodomain to mediate p53K120ac [67]. Clearly, the biochemical nature of the multi-layered and mutually exclusive modifications of the p53 C-terminus is complex. It is also puzzling that, despite association of p53 mutations with at least 50% of human cancers, Li Fraumeni syndrome is the only disease where p53 dysfunction is known to be directly involved. Adding further complexity, homozygous mice with seven lysines on p53 mutated to arginine are viable and apparently phenotypically normal [68]. Similarly, in mice with six K→R mutations in p53, expression of the protein was unaffected [69].

Since the discovery of p53 acetylation 15 years ago, numerous studies have revealed quite unexpected complexity. However, these studies also provide valuable lessons for investigating acetylation of other proteins. One realization is that, like histone acetylation, p53 acetylation does not act alone but forms an integral part of an intricate, multisite modification program. One of the strongest pieces of evidence to support the idea that the PTMs of p53 are relevant to the p53 regulatory mechanism is the fact that KDAC inhibitors have been shown to simultaneously increase the levels of acetylated p53 and induce apoptosis or senescence in cancerous and normal cells [70]. Although the PTMs of p53 are certainly important, our ability to properly identify which ones are relevant under what conditions remains rudimentary.

In addition to p53, transcription factors of the nuclear factor kappa-light-chain-enhancer of activated B cells (NF-B) family are essential regulators of the inflammatory and immune responses [71]. Acetylation of RelA/p65 by KAT3A/3B probably is associated with transcriptional activation [72,73]. Although multiple acetylation sites on p65 protein have been reported (e.g., lysine 310, 314, 315), it's the acetylation of lysine 310 that has been observed to enhance transcriptional activity without altering binding to DNA or IκB [74-76]. Acetylation of lysine 310 is blocked either in the absence of serine 276 phosphorylation or by overexpression of catalytically inactive PKAc [73]. Thus, it is speculated that phosphorylation of serine 276 on p65 triggers recruitment of KAT3A/3B that next acetylates lysine 310 on p65. It is further proposed that, in addition to p65 phosphorylation, IKKα also promotes acetylation through direct phosphorylation of N-CoR/SMRT, which displaces KDAC3 from the SMRT corepressor complex [77]. IKKα is found associated with the κB sites of NF-κB-responsive genes and stimulus-induced phosphorylation of H3 serine 10 [74,78].

In addition to cellular transcription factors, viral proteins interact to manipulate the function host's nuclear factors. It is established that control of the immune network by a human pathogenic virus starts with cooptation of the host's transcription and replication machineries. Interestingly, KATs also acetylate viral proteins; and subsequent molecular events occurring post-acetylation of viral proteins aid in control of the host's transcriptional machinery [12,79]. The best-known example is HIV transactivator protein Tat, which undergoes acetylation on lysines 28 and 50 to promote rapid replication of the HIV proviral genome [79,80]. Acetylation-mediated interactions between the cellular transcription machinery and viral proteins offer new therapeutic avenues, especially since anti-HIV drugs targeted against HIV proteins have been reported to cause drug resistance [80]. Taken together, acetylation of chromatin and transcription factors is a widespread phenomenon that not only facilitates gene regulation but also participates in numerous other cellular processes.

#### Nexus Linking Dysregulation of Metabolism and p53 Transcription Functions in Cancers

The availability of proper nutrients directly supports the synthesis of biological macromolecules that promote the growth and survival of cells and the organism. In contrast, starvation could limit cellular growth in order to sustain self-survival by using energy primarily from the breakdown of macromolecules rather than by synthesis. Clearly, metabolic pathways are tightly regulated to produce energy to allow efficient cellular growth and survival programs [81]. Evidently, tumor cells depend on metabolic changes for their continued growth and survival, and these alterations enhance the uptake of glucose and glutamine by cancer cells [82]. Therefore, components of metabolic pathways could provide new opportunities to explore potential therapeutic targets in the treatment of malignant disease. In most normal cells, the tricarboxylic acid (TCA) cycle drives the generation of ATP in the presence of oxygen (O_2_), a process known as oxidative phosphorylation. However, under conditions of limiting O_2 _or when energy is needed rapidly, glycolysis becomes the preferred route of energy production [83]. The preference of cancer cells to employ glycolysis may be a sign of response to hypoxia, which occurs as the tumor outgrows the blood supply. Although p53 can be activated during many stressors, it is speculated that hypoxia is indeed one of them. In cellular responses to hypoxia that involve the transcription factor hypoxia inducible factor (HIF), it has been shown that induction of p53 under low O_2 _concentration may trigger HIF-p53 interaction [84]. In addition, reduced nutrient or energy levels fail to stimulate both the AKT-mTOR pathway and AMP-activated protein kinase, which responds to an increased AMP/ATP ratio, resulting in p53 activation [85,86]. Furthermore, AKT activates MDM2 that regulates p53 stability; therefore, reduced AKT function will preclude MDM2 negative regulation of p53, leading to p53 activation under low-nutrient conditions. Malate dehydrogenase that converts malate to oxaloacetic acid in the TCA cycle has been shown to interact with p53 during deprivation of cellular glucose levels [87]. Okorokov and Milner noted that ADP promoted and ATP inhibited the ability of p53 to bind DNA [88]. Several studies have also documented that p53 has the capability of slowing down the glycolytic pathway to control the growth of cancerous cells by inhibiting the expression of glucose transporters [89]. Furthermore, although the underlying mechanism is still unclear, p53 can also inhibit NF-κB-mediated pro-survival pathways by limiting the activity of IκB kinase α and IκB kinase β functions [90]. Collectively, these findings indicate that a lack of nutrients and deregulation of nutrient-sensing pathways can each modulate a p53 response and that combinations of these abnormalities during tumor progression amplify the protective p53 response.

### Acetylation and regulation of metabolic enzymes

**Figure 5:**
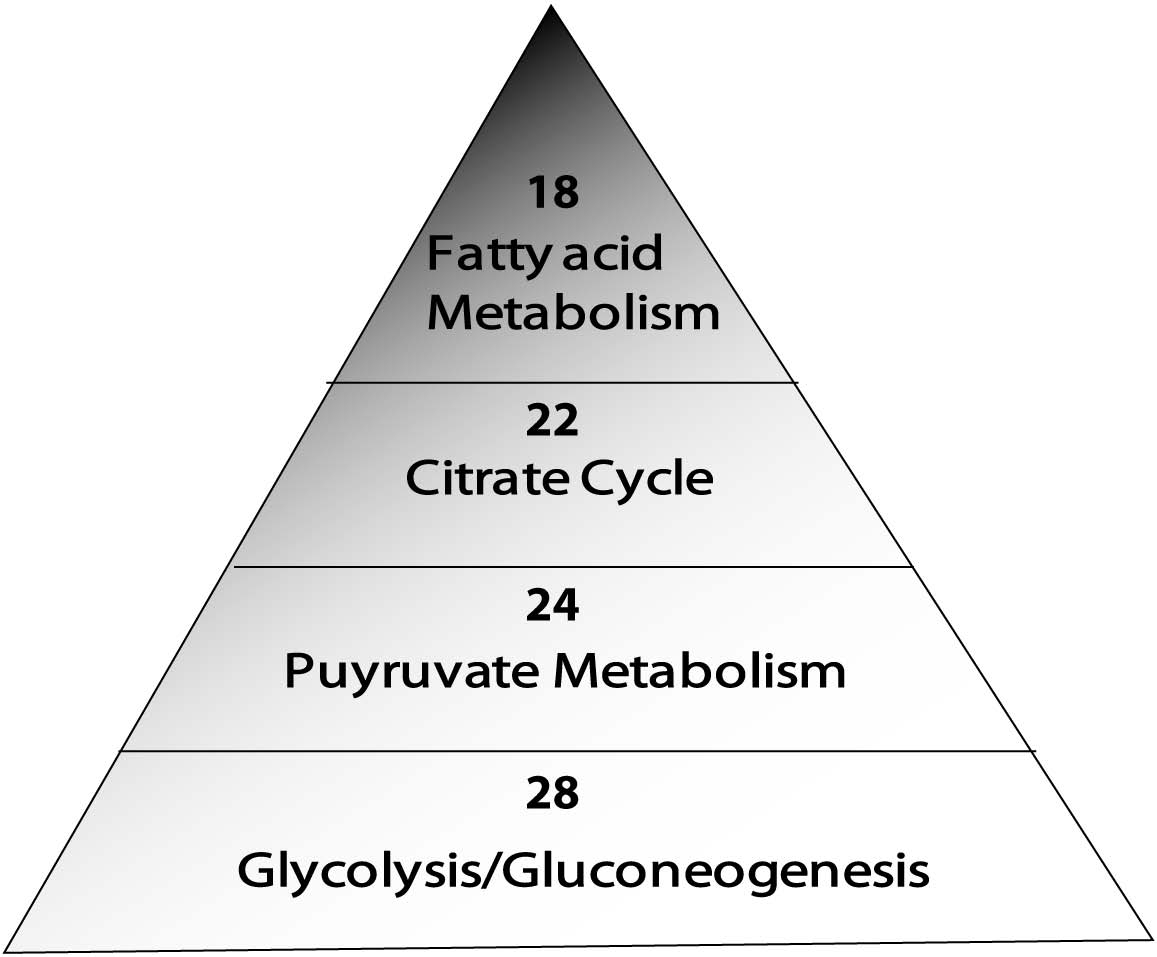
**Role of lysine acetylation in enzymes involved in intermediary metabolism**. The pyramid provides an overview of the involvement as well as overlapping roles of metabolic enzymes such as pyruvate dehydrogenase, acetyl coA synthetase and pyruvate kinase in four different metabolic pathways.

**Figure 6:**
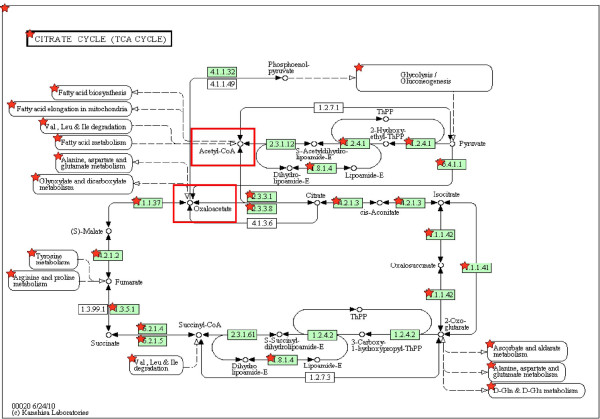
**An integrated view of the metabolic processes (fatty acid biosynthesis, glycolysis, gluconeogenesis and amino acid metabolism) that converge on the citric acid cycle**. Red stars represent lysine-acetylated metabolic enzymes and the red box highlights the critical metabolic products integrated into various metabolic pathways. Highlighted enzymes with their respective EC numbers are: pyruvate dehydrogenase (EC 1.2.4.1), dihydrolipoyl dehydrogenase (EC 1.8.1.4), pyruvate carboxylase (EC 6.4.1.1), malate dehydrogenase (EC 1.1.1.37), fumarate hydratase (EC 4.2.1.2), citrate (Si)-synthase (EC 2.3.3.1), ATP citrate synthase (EC 2.3.3.8), aconitate hydratase (EC 4.2.1.3), isocitrate dehydrogenase (NADP^+^) (1.1.1.42), isocitrate dehydrogenase (NAD^+^) (1.1.1.41), succinate dehydrogenase (1.3.5.1), succinate--CoA ligase (6.2.1.4; GDP forming), and succinate--CoA ligase (6.2.1.5; ADP forming).

Our analysis by functional clustering of the "lysine acetylome" revealed that at least 92 proteins were involved in metabolic events and energy production, including the TCA cycle, glycolysis, pyruvate metabolism, and fatty acid metabolism (Figure 5). Furthermore, as shown in Figure 6, a significant number of enzymes and the respective pathways in which they are implicated could be regulated by acetylation. For instance, 24 proteins in the TCA pathway can be acetylated (Additional file 3**Table S3**). Recently, it was reported that the activities of key enzymes regulating the choice of glycolysis versus gluconeogenesis and the branching between the TCA cycle and glyoxylate bypass could possibly be regulated by acetylation [25]. In this context, acetyl coenzyme A (Ac-coA) is particularly important owing to its unique role as the acetate donor for all cellular acetylation reactions. In mammalian cells, Ac-coA is synthesized in two pathways. In the first pathway, Ac-coA synthetase condenses acetate and coenzyme A into Ac-coA. In the second pathway, energy from hydrolyzed ATP is utilized by ATP-citrate lyase (ACL) to convert citrate, a TCA cycle intermediate, and coenzyme A into Ac-coA and oxaloacetate. Cytoplasmic Ac-coA serves as a building block for lipids, whereas nuclear Ac-coA contributes to acetylation of chromatin and its associated proteins. This demarcation within Ac-coA metabolism is necessitated by cellular need of the coenzyme. Very recently, it was demonstrated that histone acetylation in several human cell lines relies primarily on ACL activity, thus linking the TCA cycle, glycolysis and intracellular energy status to gene activity [91]. Abrogation of ACL activity results in alteration of global histone acetylation and gene transcription. Using gene knockdown strategies, it was determined that ACL is the major source of Ac-coA for histone acetylation under normal growth conditions. However, acetate supplementation following cellular deprivation of ACL is able to rescue histone acetylation, suggesting that Ac-coA production by Ac-coA synthetase can compensate for the decrease in ACL activity, which is dependent upon the availability of acetate. This mechanism may allow the acetate that is generated during histone deacetylation to be recycled back to Ac-coA. Hence, because the pool of Ac-coA for epigenetic control arises from the TCA cycle and is influenced by energy flux, the metabolic status of cells is intertwined with gene transcription. Importantly, given the continuous requirement for the high-energy compounds Ac-coA, S-adenosyl methionine (SAM) and coenzyme NAD (or NAD+) for chromatin modifications, it is of high priority to investigate whether the abundance of the latter two compounds also contributes to transcriptional regulation via epigenetic mechanisms.

Similar to Ac-coA, NAD is a key compound that captures electrons in the form of hydride during glycolysis and the TCA cycle. In contrast to many reactions in which NAD is the essential coenzyme and only undergoes a change in redox state, in sirtuin-mediated deacetylation reactions NAD is hydrolyzed into nicotinamide and *O*-acetyl-ribose. The former compound is a potent inhibitor of sirtuin KDAC activity, whereas the latter is a signaling molecule [92]. Because of the obligatory need of sirtuins for NAD in catalysis and their susceptibility to nicotinamide inhibition, the activity of sirtuins is controlled by the intracellular ratio of NAD to NADH [93,94]. During each cycle of glycolysis and TCA, in which cells derive energy from glucose and pyruvate breakdown, NAD is reduced into NADH, thus decreasing the NAD:NADH ratio that inhibits sirtuin activity. Conceptually, this reduction of sirtuin function may then be compensated for by downregulation of Ac-coA synthetase, due to its inactivation by acetylation. Fluctuation of NAD abundance modulates the activity of sirtuins that act on acetylated chromatin and transcription factors.

Histone deacetylation not only represses transcription, but also inhibits recombination. One of the functions of yeast sirtuin Sir2p is to suppress the formation of rDNA extrachromosomal circles that have been postulated to be related to cellular senescence [95]. Thus, the metabolism and availability of NAD may impact both the genome and cellular physiology in a multitude of ways, including global and local changes in nucleosomal organization and the functions of transcription factors regulated by lysine acetylation.

## Potential of targeting acetylation

The molecular events that follow acetylation could lead to recruitment of either bromodomain-containing proteins or KDACs [9,35]. Therefore, on the one hand, the enzymes that catalyze acetylation are obvious targets of intervention; on the other hand, proteins that interact with the acetylated-lysine moiety could also be potential targets [35,96]. With respect to KATs, two natural products, anacardic acid and garcinol (a polyprenylated benzophenone), are reported to inhibit both KAT3A/3B and KAT2B in the 5-10 micromolar range *in vitro *[97-99]. In contrast, curcumin displays selective activity against KAT3A/3B but not KAT2B [99]. Subsequent studies suggest that anacardic acid may be a broad-spectrum HAT inhibitor, as it also interferes with the KAT5 [100]. Isothiazolones were identified in a high-throughput screen as inhibitors of KAT2B and KAT3A/3B [101]. These compounds could be broadly useful as biological tools for evaluating the roles of HATs in transcriptional studies and may serve as lead agents for the development of novel anti-neoplastic therapeutics. Recent studies show that small molecules designed against the acetyl-lysine-binding hydrophobic pocket of conserved bromodomains affect transcriptional regulation and other cellular processes in cancer cells [102,103]. Furthermore, small-molecule modulators of KDACs have already emerged as promising therapeutic agents for cancer, cardiac illness, diabetes, and neurodegenerative disorders. Hence, studies focusing on lysine acetylation as well as molecular events that follow acetylation could identify non-histone targets for KAT- and KDAC-modulating compounds as well as illuminate molecular basis of signaling on chromatin and unravel new avenues to improve the efficacy of related therapeutic agents.

## Conclusions and future perspectives

Evidently, many acetylated proteins are not only key components within nuclear processes, but also play crucial roles in signaling pathways, such as DNA damage response, immune network and inflammation. This has propelled the idea that instead of phosphorylation being the major contributor, signaling pathways are possibly controlled by the synchronized actions of phosphorylation, acetylation, and several other PTMs. Although acetylation regulates the activity of metabolic enzymes, the role of phosphorylation in conjunction with acetylation in metabolic pathways is not clear. However, what is clear is that lysine acetylation definitely expands the plasticity within the metabolic and cellular signaling networks. This notion is reinforced by the recent analyses of the "lysine acetylome" explicated above, which broadened the scope of acetylation-mediated regulation through an expansive clustering into diverse functional groups. This list offers new insights into the role of acetylation and possible routes to dissect its mechanism, especially in regulation of diseases like cancer and neurodegenerative disorders.

Meanwhile, proteomic surveys by MS will continue to identify new acetylated proteins, which along with efficient mapping of acetylation sites by MS should reveal additional sites [104]. For instance, accumulating studies on p53 acetylation indicate that, subsequent to *in vitro *biochemical characterization, cell lines and genetically altered mouse models will be especially effective for characterizing the biological functions associated with particular lysine PTMs [105]. Most importantly, such approaches will facilitate mapping within signaling pathways that are regulated by reversible acetylation. One pertinent question is how such modifications interact with other PTMs within the same or different protein(s) and form dynamic programs for regulating cellular functions under normal and pathological settings. Within the acetylproteome, the functional impact of lysine acetylation is context-dependent and varies from protein to protein. As in histones, the molecular interplay of lysine acetylation with other PTMs, either agonistically or antagonistically, generates codified molecular signaling programs that are crucial for governing the functions of various nuclear and cytoplasmic proteins [9,106-108].

## Competing interests

The authors declare that they have no competing interests.

## Authors' contributions

JP initiated the project and participated in the design of the project. RRP performed the bioinformatics analysis. SM conceived the idea, coordinated the project and drafted the manuscript. All authors have read and have approved the manuscript.

## Supplementary Material

Additional file 1 **Functional clusters were generated from the list of lysine-acetylated proteins using the DAVID 6.7 bioinformatics resources functional annotation tool**. The acetylated proteins are distributed into 526 functional clusters generated by the software using a heuristic fuzzy clustering concept that measures relationships among the annotation terms based on the degree of their co-association to cluster similar annotations into functional annotation groups. The annotation clusters are assigned "enrichment scores" in decreasing order of occurrence that are quantitatively measured by some common and well-known statistical methods, including Fisher's exact test, binomial probability and hypergeometric distribution. Click here for file

Additional file 2 **Lysine-acetylated proteins involved in different forms of cancers**. The list of proteins includes heat-shock family members, kinases and transcription factors. Click here for file

Additional file 3 **List of lysine-acetylated proteins associated with enzymes involved in metabolic pathways**. Click here for file
